# A Note on Decomposing a Square Matrix as Sum of Two Square Nilpotent Matrices over an Arbitrary Field

**DOI:** 10.1155/2013/640350

**Published:** 2013-11-11

**Authors:** Xiaofei Song, Baodong Zheng, Chongguang Cao

**Affiliations:** ^1^Department of Mathematics, Harbin Institute of Technology, Harbin 150001, China; ^2^School of Mathematical Sciences, Heilongjiang University, Harbin 150080, China

## Abstract

Let *K* be an arbitrary field and *X* a square matrix over *K*. Then *X* is sum of two square nilpotent matrices over *K* if and only if, for every algebraic extension *L* of *K* and arbitrary nonzero *α* ∈ *L*, there exist idempotent matrices *P*_1_ and *P*_2_ over *L* such that *X* = *αP*_1_ − *αP*_2_.

## 1. Introduction

 Botha (see [1]) proved that a square matrix *A* over a field *K* is a sum of two nilpotent matrices over *K* if and only if *A* is similar to a particular form. In an early paper, Pazzis (see [2]) gave necessary and sufficient conditions in which a matrix can be decomposed as a linear combination of two idempotents with given nonzero coefficients. The goal of this paper is to build a bridge that connects the result obtained in [1] with the result obtained in [2]. However, the relation between these two facts has not been formally discussed yet (more details in [3–9]).

If there is no statement, the meanings of notations mentioned in this paragraph hold all over the paper. *K* denotes an arbitrary field, $\bar{K}$ is its algebraic closure, *L* is an arbitrary algebraic extension of *K*, and car(*K*) is the characteristic of *K*. *Z* ^+^ denotes the set of all positive integers, [*s*] = {*z* ∈ *Z* ^+^ | 1 ≤ *z* ≤ *s*} for some *s* ∈ *Z* ^+^. *M*_*m*,*n*_(*K*) denotes the space consisting of all *m* × *n* matrices over *K*; *M*_*n*_(*K*) = *M*_*n*,*n*_(*K*). *r*(*A*) is the rank of *A* ∈ *M*_*m*,*n*_(*K*). *E* denotes a vector space over *K* and dim⁡(*E*) is the dimension of *E*. *X* ∈ *M*_*n*_(*K*) is called _*s*_^2^ *N* in *M*_*n*_(*K*) if there exist square nilpotent *N*_1_ and *N*_2_ ∈ *M*_*n*_(*K*) such that *X* = *N*_1_ + *N*_2_, while *X* is called an (*α*, *β*) composite in *M*_*n*_(*K*) if there exist idempotent *P*_1_ and *P*_2_ ∈ *M*_*n*_(*K*) such that *X* = *αP*_1_ + *βP*_2_, where *α*, *β* ∈ *K*∖{0} (Definition 1 in [2]); in particular, *X* is called _±_ *P* if *X* is an (*α*, −*α*) composite in *M*_*n*_(*L*) for every algebraic extension *L* of *K* and arbitrary nonzero *α* ∈ *L* (when car(*K*) = 2, we still use _±_ *P* for the meaning of (*α*, *α*) composites).

For *X* ∈ *M*_*n*_(*K*), on the one hand, we will prove that *X* is _*s*_^2^ *N* in *M*_*n*_(*K*) implies *X* is _±_ *P*; that is, the form provided by Botha satisfies the condition as in [2]; on the other hand, we will also prove that *X* is _±_ *P* implies *X* is _*s*_^2^ *N* in *M*_*n*_(*K*); that is, we can derive the form provided in [1] from the results obtained in [2]. In fact, the following theorem is the main result of this paper.

**Theorem 1 (main theorem)**

Suppose *K* is an arbitrary field and *X* ∈ *M*_*n*_(*K*); then *X* is _*s*_^2^ *N* in *M*_*n*_(*K*) if and only if *X* is _±_ *P*.

In Section 2, we will state some related theorems and notations from [2] and we will give some necessary corollaries. The proof of Theorem 1 will be carried out in Section 3.

## 2. More Notations and Necessary Corollaries

Suppose *X* ∈ *M*_*n*_(*K*) and *X*_*i*_ ∈ *M*_*n*_*i*__(*K*), we denote by *X* = *X*_1_ ⊕ ⋯⊕*X*_*s*_ the following matrix with ∑_*i*=1_^*s*^ *n*_*i*_ = *n*:

(1) $$\begin{array}{c} \left(\begin{array}{cccc} X_{1} & & & \\ & X_{2} & & \\ & & \ddots & \\ & & & X_{s} \end{array}\right). \end{array}$$

**Notation 1 (Notation 2 in [2])**

Let *X* ∈ *M*_*n*_(*K*), $\lambda \in \bar{K}$ and *k* ∈ *Z* ^+^; we denote by

1. *j*_*k*_(*X*, *λ*) the number of blocks of size *k* for the eigenvalue *λ* in the Jordan reduction of *X*;
2. *n*_*k*_(*X*, *λ*) the number of blocks of size greater or equal to *k* for the eigenvalue *λ* in the Jordan reduction of *X*.

**Definition 2 (Definition 3 in [2])**

Two sequences (*u*_*k*_)_*k*  ≥1_ and (*v*_*k*_)_*k*  ≥1_ are side to be intertwined if for  all  *k* ∈ *Z* ^+^, *v*_*k*_ ≥ *u*_*k*+1_, and *u*_*k*_ ≥ *v*_*k*+1_.

**Notation 2 (Notation 4 in [2])**

Given a monic polynomial, *P* = *x* ^*n*^ − *a*_*n*−1_ *x* ^*n*−1^ − ⋯−*a*_1_ *x* − *a*_0_, denote the following *C*(*P*) by its companion matrix:

(2) $$\begin{array}{c} C\left(P\right)=\left(\begin{array}{cccccc} 0 & 0 & \cdots & \cdots & 0 & a_{0}\\ 1 & 0 & 0 & \cdots & 0 & a_{1}\\ 0 & 1 & 0 & \ddots & 0 & a_{2}\\ \vdots & \ddots & \ddots & \ddots & \ddots & \vdots \\ \vdots & \cdots & \ddots & 1 & 0 & a_{n-2}\\ 0 & \cdots & \cdots & 0 & 1 & a_{n-1} \end{array}\right). \end{array}$$

**Theorem 3 (Theorem 1 in [2])**

Assume car (*K*) ≠ 2 and let *X* ∈ *M*_*n*_(*K*). Then *X* is an (*α*, −*α*) composite if and only if all the following conditions hold.

1. The sequences (*n*_*k*_(*X*, *α*))_*k*  ≥1_ and (*n*_*k*_(*X*, −*α*))_*k*  ≥1_ are intertwined;
2. $\text{for}\;\text{all}\;\lambda \in \bar{K}\setminus \{0,\alpha ,-\alpha \}$ and for all *k* ∈ *Z* ^+^, *j*_*k*_(*X*, *λ*) = *j*_*k*_(*X*, −*λ*).

**Theorem 4 (Theorem 5 in [2])**

Assume car(*K*) = 2 and let *X* ∈ *M*_*n*_(*K*). Then *X* is an (*α*, −*α*) composite if and only if for every $\lambda \in \bar{K}\setminus \{0,\alpha \}$, all blocks in the Jordan reduction of *X* with respect to *λ* have an even size.

Suppose *X* ∈ *M*_*n*_(*k*) is _±_ *P*, where car(*K*) ≠ 2. Then *X* is (*α*, −*α*) composite and (*β*, −*β*) composite in *M*_*n*_(*L*) for some algebraic extension *L* of *K*, where *α*, *β* ∈ *L*∖{0} with *α* ≠ ±*β*. By Theorem 3, the following statements are true:

1. $\text{for}\;\text{all}\;\lambda \in \bar{K}\in \{0,\alpha ,-\alpha \}$ and for all *k* ∈ *Z* ^+^, *j*_*k*_(*X*, *λ*) = *j*_*k*_(*X*, −*λ*);
2. $\text{for}\;\text{all}\;\lambda \in \bar{K}\in \left\{0,\beta ,-\beta \right\}\;\text{and}\;\text{for}\;\text{all}\;k\in Z^{+}$, *j*_*k*_(*X*, *λ*) = *j*_*k*_(*X*, −*λ*).

so $\text{for}\;\text{all}\;\lambda \in \bar{K}\setminus \{0\}$ and for  all  *k* ∈ *Z* ^+^, *j*_*k*_(*X*, *λ*) = *j*_*k*_(*X*, −*λ*).

On the other hand, note that for nonzero $\alpha \in \bar{K}$ with car(*K*) ≠ 2, the sequences (*n*_*k*_(*X*, *α*))_*k*  ≥1_ and (*n*_*k*_(*X*, −*α*))_*k*  ≥1_ are intertwined if for  all  *k* ∈ *Z* ^+^, *j*_*k*_(*X*, *α*) = *j*_*k*_(*X*, −*α*). Then $\text{for}\;\text{all}\;\lambda \in \bar{K}\setminus \{0\}$, *k* ∈ *Z* ^+^, *j*_*k*_(*X*, *λ*) = *j*_*k*_(*X*, −*λ*) implies that for every algebraic extension *L* of *K* and arbitrary nonzero *α* ∈ *L*, *X* is an (*α*, −*α*) composite in *M*_*n*_(*L*); that is, *X* is _±_ *P*.

Therefore the following corollary is true.

**Corollary 5**

Assume car(*K*) ≠ 2 and let *X* ∈ *M*_*n*_(*K*). Then *X* is _±_ *P* if and only if $\text{ for}\;\text{all }\;\lambda \in \bar{K}\setminus \left\{0\right\}\;\text{ for}\;\text{all }\;k\in Z^{+}$, *j*_*k*_(*X*, *λ*) = *j*_*k*_(*X*, −*λ*).

Similarly, we can derive the following corollary from Theorem 4.

**Corollary 6**

Assume car(*K*) = 2 and let *X* ∈ *M*_*n*_(*K*). Then *X* is_±_ *P* if and only if for every $\lambda \in \bar{K}\setminus \{0\}$, all blocks in the Jordan reduction of *X* with respect to *λ* have an even size.

Naturally, we derive the following corollary from the above two corollaries.

**Corollary 7**

Every nilpotent is _±_ *P*.

In fact, arbitrary nilpotent is not only _±_ *P* but also _*s*_^2^ *N*.

**Lemma 8**

Every nilpotent *N* ∈ *M*_*n*_(*K*) is _*s*_^2^ *N*.

**Proof**

For arbitrary field *K*, let *N* ∈ *M*_*n*_(*K*) is nilpotent; then *N* is similar to *N*_1_ ⊕ *N*_2_ ⊕ ⋯⊕*N*_*s*_, where for every *i* ∈ [*s*], *N*_*i*_ ∈ *M*_*r*_*i*__(*K*), ∑_*i*=1_^*s*^ *r*_*i*_ = *n*, and both the characteristic polynomial and minimal polynomial of *N*_*i*_ are *x* ^*r*_*i*_^. Furthermore, *N*_*i*_ is similar to *C*(*x* ^*r*_*i*_^) as follows:

(3) $$\begin{array}{c} {\left(\begin{array}{cccc} 0 & 0 & \cdots & 0\\ 1 & 0 & \cdots & 0\\ \vdots & \ddots & \ddots & \vdots \\ 0 & \cdots & 1 & 0 \end{array}\right)}_{r_{i}\times r_{i}}. \end{array}$$

That is, *C*(*x* ^*r*_*i*_^) = *E*_2,1_ + *E*_3,2_ + ⋯+*E*_*r*_*i*_,*r*_*i*_−1_ ∈ *M*_*r*_*i*__(*K*).

When *r*_*i*_ is even, *C*(*x* ^*r*_*i*_^) = ∑_*j*=1_^*r*_*i*_/2^ *E*_2*j*,2*j*−1_ + ∑_*j*=1_^*r*_*i*_/2−1^ *E*_2*j*+1,2*j*_; when *r*_*i*_ is odd, *C*(*x* ^*r*_*i*_^) = ∑_*j*=1_^(*r*_*i*_−1)/2^ *E*_2*j*,2*j*−1_ + ∑_*j*=1_^(*r*_*i*_−1)/2^ *E*_2*j*+1,2*j*_. Note that both ∑*E*_2*j*,2*j*−1_ and ∑*E*_2*j*+1,2*j*_ are square nilpotent matrices then *C*(*x* ^*r*_*i*_^) is _*s*_^2^ *N*, and *N*_*i*_ is _*s*_^2^ *N* follows. Hence *N* is _*s*_^2^ *N*.

## 3. Proof of Main Theorem

_*s*_^2^ *N* → _±_ *P*. Suppose *X* ∈ *M*_*n*_(*K*) is _*s*_^2^ *N* in *M*_*n*_(*K*); that is, there exist square nilpotent matrices *N*_1_ and *N*_2_ ∈ *M*_*n*_(*K*) such that *X* = *N*_1_ + *N*_2_. It will take two steps to prove *X* is _±_ *P*.

**Step 1**

If *X* is nonsingular, then *X* is _±_ *P*.

Since *X* = *N*_1_ + *N*_2_ with *N*_1_^2^ = *N*_2_^2^ = 0, inspect the eigenspaces of *N*_1_ and *N*_2_. Note that *N*_1_ and *N*_2_ are square nilpotent matrices, their ranks satisfy the following inequality matrices.

(4) $$\begin{array}{c} r\left(N_{1}\right)+r\left(N_{2}\right)\leq n, \end{array}$$

where equality holds if and only if *r*(*N*_1_) = *r*(*N*_2_) = *n*/2.

At first, *X* is nonsingular implies 0 is not its eigenvalue. Secondly, if the inequality is strict, then intersection of eigenspaces of *N*_1_ and *N*_2_ contains nonzero vectors; that is, there exists nonzero *x* ∈ *M*_*n*,1_(*K*) such that *N*_1_ *x* = *N*_2_ *x* = 0, which implies that 0 is one of eigenvalues of *X*. This is a contradiction. Hence, *r*(*N*_1_) + *r*(*N*_2_) = *n*; that is, *n* is even and *N*_1_ and *N*_2_ are similar but not equal.

Because *N*_1_ is square nilpotent with *r*(*N*_1_) = *n*/2, we can choose *n*/2 linear independent vectors from the set of its column vectors which can make up a base of eigenspace of *N*_1_ and denote *β* by the *n* × (*n*/2) matrix consisting of these *n*/2 columns. Correspondingly, we have *n* × (*n*/2) matrix *γ* with all columns from the set of columns of *N*_2_. Because 0 is the only vector in the intersection of eigenspaces of *N*_1_ and *N*_2_, *n* × *n* matrix $\left(\begin{array}{cc} \beta & \gamma \end{array}\right)$ is nonsingular.

*N*_1_^2^ *γ* = 0 implies that nonzero column vectors of *N*_1_ *γ* are eigenvectors of *N*_1_ and $N_{1}\left(\begin{array}{cc} \beta & \gamma \end{array}\right)=\left(\begin{array}{cc} 0 & N_{1}\gamma \end{array}\right)$ implies *r*(*N*_1_ *γ*) = *n*/2. Hence; *N*_1_ *γ* and *β* are equal under certain column transformation; that is, there is an invertible matrix *T*_1_ such that *N*_1_ *γ* = *βT*_1_. Correspondingly, there is an invertible matrix *T*_2_ such that *N*_2_ *β* = *γT*_2_.

Let $\left(\begin{array}{c} y_{1}\\ y_{2} \end{array}\right)$ be the inverse of $\left(\begin{array}{cc} \beta & \gamma \end{array}\right)$, where *y*_1_ and *y*_2_ are (*n*/2) × *n* matrices. Naturally, the following equation is true:

(5) $$\begin{array}{c} \left(\begin{array}{c} y_{1}\\ y_{2} \end{array}\right)\left(\begin{array}{cc} \beta & \gamma \end{array}\right)=\left(\begin{array}{cc} y_{1}\beta & y_{1}\gamma \\ y_{2}\beta & y_{2}\gamma \end{array}\right)=\left(\begin{array}{cc} I_{n/2} & 0_{n/2}\\ 0_{n/2} & I_{n/2} \end{array}\right). \end{array}$$

Now, we carry out the same similarity transformation on *N*_1_ and *N*_2_ as follows:

(6) $$\begin{array}{c} \left(\begin{array}{c} y_{1}\\ y_{2} \end{array}\right)N_{1}\left(\begin{array}{cc} \beta & \gamma \end{array}\right)=\left(\begin{array}{cc} y_{1}N_{1}\beta & y_{1}N_{1}\gamma \\ y_{2}N_{1}\beta & y_{2}N_{1}\gamma \end{array}\right),\\ \left(\begin{array}{c} y_{1}\\ y_{2} \end{array}\right)N_{2}\left(\begin{array}{cc} \beta & \gamma \end{array}\right)=\left(\begin{array}{cc} y_{1}N_{2}\beta & y_{1}N_{2}\gamma \\ y_{2}N_{2}\beta & y_{2}N_{2}\gamma \end{array}\right). \end{array}$$

Note that *N*_1_ *γ* = *βT*_1_ and *N*_2_ *β* = *γT*_2_, the above three equations imply that *N*_1_ is similar to $\left(\begin{array}{cc} 0_{n/2} & T_{1}\\ 0_{n/2} & 0_{n/2} \end{array}\right)\in M_{n}(K)$ and *N*_2_ is similar to $\left(\begin{array}{cc} 0_{n/2} & 0_{n/2}\\ T_{2} & 0_{n/2} \end{array}\right)\in M_{n}(K)$.

Hence, *X* is similar to $\left(\begin{array}{cc} 0_{n/2} & T_{1}\\ 0_{n/2} & 0_{n/2} \end{array}\right)+\left(\begin{array}{cc} 0_{n/2} & 0_{n/2}\\ T_{2} & 0_{n/2} \end{array}\right)$. For every algebraic extension *L* of *K* and arbitrary nonzero *α* ∈ *L*, *X* is also similar to the following matrix:

(7) $$\begin{array}{c} \alpha \left(\begin{array}{cc} I_{n/2} & \alpha^{-1}T_{1}\\ 0_{n/2} & 0_{n/2} \end{array}\right)-\alpha \left(\begin{array}{cc} I_{n/2} & 0_{n/2}\\ -\alpha^{-1}T_{2} & 0_{n/2} \end{array}\right) \end{array}$$

That is, *X* is _±_ *P*.

**Step 2**

If *X* is singular and similar to *Y* ⊕ *N*, where *Y* is nonsingular and *N* is nilpotent. Then *X* is _±_ *P*.

At first, we need to prove that *Y* is _*s*_^2^ *N*. Without loss of generality, we assume *X* = *Y* ⊕ *N* in the following proof since _*s*_^2^ *N* holds under similarity transformations.

Let $N_{1}=\left(\begin{array}{cc} n_{1} & n_{2}\\ n_{3} & n_{4} \end{array}\right)$, where the order of *n*_1_ is the same for *Y* and the order of *n*_4_ is the same for *N*. Then *N*_1_^2^ = 0 implies the following equations are true:

(8) $$\begin{array}{c} n_{1}^{2}+n_{2}n_{3}=0,\;\;n_{1}n_{2}+n_{2}n_{4}=0\\ n_{3}n_{1}+n_{4}n_{3}=0,\;\;n_{3}n_{2}+n_{4}^{2}=0. \end{array}$$

Since (*X* − *N*_1_)^2^ = *N*_2_^2^ = 0, we get the following equations after replacing *n*_1_ with *Y* − *n*_1_ and *n*_4_ with *N* − *n*_4_ in the previous equations:

(9) $$\begin{array}{c} {\left(Y-n_{1}\right)}^{2}+n_{2}n_{3}=0,\\ \left(Y-n_{1}\right)n_{2}+n_{2}\left(N-n_{4}\right)=0,\\ n_{3}\left(Y-n_{1}\right)+\left(N-n_{4}\right)n_{3}=0,\\ n_{3}n_{2}+{\left(N-n_{4}\right)}^{2}=0. \end{array}$$

We can derive the following equations from the 3rd and 4th equations in the above two sets of equations:

(10) $$\begin{array}{c} Yn_{2}+n_{2}N=0,\;\;\;n_{3}Y+Nn_{3}=0. \end{array}$$

Note that *N* is nilpotent, assume its index is *r*; that is, *N* ^*r*−1^ ≠ 0 and *N* ^*r*^ = 0. After multiplying the right side of equation *Yn*_2_ + *n*_2_ *N* = 0 by *N* ^*r*−1^, we can get *Yn*_2_ *N* ^*r*−1^ = 0. *Y* is nonsingular implies *n*_2_ *N* ^*r*−1^ = 0. Repeat the operation, we eventually get *n*_2_ = 0. Similarly, we can also get *n*_3_ = 0.

So *N*_1_ is quasidiagonal and *N*_2_ is also quasidiagonal through similar proof; that is, *n*_1_ and *n*_4_ are square nilpotent same as the corresponding parts of *N*_2_. Finally, we prove that *Y* is _*s*_^2^ *N*.

Since *Y* is _±_ *P* by Step 1 and *N* is _±_ *P* by Corollary 7, it is true that *X* is _±_ *P*.

_±_ *P*→_*s*_^2^ *N*. Suppose *X* ∈ *M*_*n*_(*K*) is _±_ *P*. If *X* is similar to *Y* ⊕ *N*, where *Y* is nonsingular and *N* is nilpotent, then *X* is _±_ *P* if and only if *Y* is _±_ *P* by Corollaries 5, 6, and 7. Without loss of generality, we can assume *X* is nonsingular. Furthermore, if *X* is nonsingular and similar to *Y*_1_ ⊕ *Y*_2_, where all eigenvalues of *Y*_1_ are not in *K* and all eigenvalues of *Y*_2_ are in *K*. Then *X* is _±_ *P* if and only if *Y*_1_ is _±_ *P* and *Y*_2_ is _±_ *P*. It will take two steps to prove *X* is _*s*_^2^ *N*.

**Step 3**

Suppose car(*K*) ≠ 2 and all eigenvalues of *X* are not in *K*; then for arbitrary nonzero *α* ∈ *K*, *X* is an (*α*, −*α*) composite; that is, there exist idempotent matrices *P*_1_ and *P*_2_ ∈ *M*_*n*_(*K*) such that *X* = *αP*_1_ − *αP*_2_.

Let *Q*_1_^(0)^ be the eigenspace of *P*_1_ with respect to 0, *Q*_1_^(1)^ the eigenspace of *P*_1_ with respect to 1, let *Q*_2_^(0)^ be the eigenspace of *P*_2_ with respect to 0, and *Q*_2_^(1)^ the eigenspace of *P*_2_ with respect to 1. Both *α* and −*α* are not eigenvalues of *X* implies that *Q*_1_^(0)^∩*Q*_2_^(0)^ = *Q*_1_^(1)^∩*Q*_2_^(1)^ = *Q*_1_^(0)^∩*Q*_2_^(1)^ = *Q*_1_^(1)^∩*Q*_2_^(0)^ = {0}; then dim⁡(*Q*_1_^(0)^) = dim⁡(*Q*_1_^(1)^) = dim⁡(*Q*_2_^(0)^) = dim⁡(*Q*_2_^(1)^) = *n*/2 (otherwise, dim⁡(*Q*_1_^(0)^) ≥ *n*/2 implies *Q*_1_^(0)^∩*Q*_2_^(0)^ ≠ {0} or *Q*_1_^(0)^∩*Q*_2_^(1)^ ≠ {0}, etc.); that is, *n* is even.

Suppose *S* and *T* are *n* × (*n*/2) matrices with *r*(*S*) = *r*(*T*) = *n*/2 satisfying *P*_1_ *S* = 0 and *P*_2_ *T* = *T*; then $\left(\begin{array}{cc} S & T \end{array}\right)$ is *n* × *n* nonsingular matrix. Let $\left(\begin{array}{c} U\\ V \end{array}\right)$ be its inverse; that is,

(11) $$\begin{array}{c} \left(\begin{array}{c} U\\ V \end{array}\right)\left(\begin{array}{cc} S & T \end{array}\right)=\left(\begin{array}{cc} US & UT\\ VS & VT \end{array}\right)=\left(\begin{array}{cc} I_{n/2} & 0_{n/2}\\ 0_{n/2} & I_{n/2} \end{array}\right). \end{array}$$

Then we carry out the same similarity transformation on *P*_1_ and *P*_2_ as follows:

(12) $$\begin{array}{c} \left(\begin{array}{c} U\\ V \end{array}\right)P_{1}\left(\begin{array}{cc} S & T \end{array}\right)=\left(\begin{array}{cc} UP_{1}S & UP_{1}T\\ VP_{1}S & VP_{1}T \end{array}\right)=\left(\begin{array}{cc} 0_{n/2} & UP_{1}T\\ 0_{n/2} & VP_{1}T \end{array}\right),\\ \left(\begin{array}{c} U\\ V \end{array}\right)P_{2}\left(\begin{array}{cc} S & T \end{array}\right)=\left(\begin{array}{cc} UP_{2}S & UP_{2}T\\ VP_{2}S & VP_{2}T \end{array}\right)=\left(\begin{array}{cc} UP_{2}S & 0_{n/2}\\ VP_{2}S & I_{n/2} \end{array}\right), \end{array}$$

where *P*_1_ and *P*_2_ are idempotent implies that *VP*_1_ *T* and *UP*_2_ *S* are idempotent and *r*(*P*_1_) = *r*(*P*_2_) = *n*/2 implies that *VP*_1_ *T* = *I*_*n*/2_ and *UP*_2_ *S* = 0_*n*/2_. Hence, *X* is similar to the following matrix:

(13) $$\begin{array}{c} \left(\begin{array}{cc} 0_{n/2} & \alpha UP_{1}T\\ 0_{n/2} & 0_{n/2} \end{array}\right)+\left(\begin{array}{cc} 0_{n/2} & 0_{n/2}\\ -\alpha VP_{2}S & 0_{n/2} \end{array}\right). \end{array}$$

That is, *X* is _*s*_^2^ *N* in *M*_*n*_(*K*).

When car(*K*) = 2, *X* is (*α*, *α*) composite for arbitrary nonzero *α* ∈ *K*, we can similarly prove that *X* is _*s*_^2^ *N* in *M*_*n*_(*K*) replacing −*α* with *α* in the previous proof.

**Step 4**

Suppose car(*K*) ≠ 2 and all eigenvalues of *X* are in *K*; then by Corollary 5, *j*_*k*_(*X*, *α*) = *j*_*k*_(*X*, −*α*) for every *k* ∈ *Z* ^+^ and arbitrary nonzero *α* ∈ *K*.

Moreover, *X* is similar to *X*_1_ ⊕ ⋯⊕*X*_*s*_, where both the characteristic polynomial and the minimal polynomial of *X*_*i*_ are [(*x* − *α*_*i*_)(*x* + *α*_*i*_)]^*r*_*i*_^ = (*x* ^2^ − *α*_*i*_^2^)^*r*_*i*_^ with 2∑_*i*=1_^*s*^ *r*_*i*_ = *n* and *α*_*i*_ ∈ *K*∖{0} is one of eigenvalues of *X* for every *i* ∈ [*s*]. Without loss of generality, we just need to prove *X*_*i*_ is _*s*_^2^ *N*.

Since *X*_*i*_ is similar to *C*((*x* ^2^ − *α*_*i*_^2^)^*r*_*i*_^) as follows:

(14) $$\begin{array}{c} \left(\begin{array}{cccccc} 0 & 0 & \cdots & \cdots & 0 & a_{0}\\ 1 & 0 & \cdots & \cdots & 0 & 0\\ 0 & 1 & 0 & \cdots & 0 & a_{2}\\ \vdots & \ddots & \ddots & \ddots & \vdots & \vdots \\ \vdots & \ddots & \ddots & 1 & 0 & a_{2r_{i}-2}\\ 0 & \cdots & \cdots & 0 & 1 & 0 \end{array}\right), \end{array}$$

where (*x* ^2^ − *α*_*i*_^2^)^*r*_*i*_^ = *x* ^2*r*_*i*_^ − *a*_2*r*_*i*_−2_ *x* ^2*r*_*i*_−2^ − ⋯−*a*_2_ *x* ^2^ − *a*_0_. We have *C*((*x* ^2^ − *α*_*i*_^2^)^*r*_*i*_^) = *E*_2,1_ + ⋯+*E*_2*r*_*i*_,2*r*_*i*_−1_ + *a*_0_ *E*_1,2*r*_*i*__ + *a*_2_ *E*_3,2*r*_*i*__ + ⋯+*a*_2*r*_*i*_−2_ *E*_2*r*_*i*_−1,2*r*_*i*__ = (*E*_2,1_ + *E*_4,3_ + ⋯+*E*_2*r*_*i*_,2*r*_*i*_−1_)+(*E*_3,2_ + ⋯+*E*_2*r*_*i*_−1,2*r*_*i*_−2_ + *a*_0_ *E*_1,2*r*_*i*__ + *a*_2_ *E*_3,2*r*_*i*__ + ⋯+*a*_2*r*_*i*_−2_ *E*_2*r*_*i*_−1,2*r*_*i*__) = *N*_1_ + *N*_2_. Obviously, both *N*_1_ and *N*_2_ are square nilpotent matrices; that is, *X*_*i*_ is _*s*_^2^ *N*. Hence, *X* is _*s*_^2^ *N* in *M*_*n*_(*K*).

When car(*K*) = 2, all blocks in the Jordan reduction of *X* with respect to *α* ∈ *K*∖{0} have an even size by Corollary 6; that is, both the characteristic polynomial and minimal polynomial of every block with respect to *α* are (*x* + *α*)^*s*_*i*_^ = ((*x* + *α*)^2^)^*s*_*i*_/2^ = (*x* ^2^ + *α* ^2^)^*s*_*i*_/2^, where *s*_*i*_ is even. Similarly, we can also prove that *X* is _*s*_^2^ *N* in *M*_*n*_(*K*).
